# Gaze Tracking System for User Wearing Glasses

**DOI:** 10.3390/s140202110

**Published:** 2014-01-27

**Authors:** Su Yeong Gwon, Chul Woo Cho, Hyeon Chang Lee, Won Oh Lee, Kang Ryoung Park

**Affiliations:** Division of Electronics and Electrical Engineering, Dongguk University, 26 Pil-dong 3-ga, Jung-gu, Seoul 100-715, Korea; E-Mails: gwonsuyeong@dongguk.edu (S.Y.G.); cho4400@dongguk.edu (C.W.C.); leehc@dongguk.edu (H.C.L.); 215p8@hanmail.net (W.O.L.)

**Keywords:** gaze tracking, glasses, controlling device for illuminator, pupil and corneal specular reflection

## Abstract

Conventional gaze tracking systems are limited in cases where the user is wearing glasses because the glasses usually produce noise due to reflections caused by the gaze tracker's lights. This makes it difficult to locate the pupil and the specular reflections (SRs) from the cornea of the user's eye. These difficulties increase the likelihood of gaze detection errors because the gaze position is estimated based on the location of the pupil center and the positions of the corneal SRs. In order to overcome these problems, we propose a new gaze tracking method that can be used by subjects who are wearing glasses. Our research is novel in the following four ways: first, we construct a new control device for the illuminator, which includes four illuminators that are positioned at the four corners of a monitor. Second, our system automatically determines whether a user is wearing glasses or not in the initial stage by counting the number of white pixels in an image that is captured using the low exposure setting on the camera. Third, if it is determined that the user is wearing glasses, the four illuminators are turned on and off sequentially in order to obtain an image that has a minimal amount of noise due to reflections from the glasses. As a result, it is possible to avoid the reflections and accurately locate the pupil center and the positions of the four corneal SRs. Fourth, by turning off one of the four illuminators, only three corneal SRs exist in the captured image. Since the proposed gaze detection method requires four corneal SRs for calculating the gaze position, the unseen SR position is estimated based on the parallelogram shape that is defined by the three SR positions and the gaze position is calculated. Experimental results showed that the average gaze detection error with 20 persons was about 0.70° and the processing time is 63.72 ms per each frame.

## 1. Introduction

Recently, human biometric information has been used widely in various applications. Hand motions, finger shape information, and the head movements of users can be used as inputs for devices in various systems [1,2]. Texture information from the iris and the face of a user can be used for security systems [3,4]. Gaze tracking technology, which is based on the eye positions of users, has been highlighted because of the potential that it holds for natural user interfaces. This technology can be used to create intelligent interfaces with TVs and computers [5–8].

Conventional gaze tracking systems calculate the gaze position by detecting the center of the pupil and detecting the corneal specular reflection (SR) points that are produced by illuminators. Therefore, accurate detections of the locations of the pupil center and the corneal SRs are crucial for achieving high levels of accuracy during the gaze estimation process [8–12]. However, when a user is wearing glasses, the illuminators of the gaze tracking system typically produce a lot of reflections and noise from the surfaces of glasses. These reflections can hide the corneal SRs or the pupil in the image of the eye, which in turn reduces the accuracy of the gaze detection process.

The shape and size of these reflections vary based on the level of cleanliness of the glass surfaces, the power of the glasses, and the user's head movements. Previous studies have taken the illumination noises from glasses into consideration during the gaze position detection process for users who were wearing glasses [12–15]. Ying *et al.*, proposed a method based on a pyramidal multi-scale screening algorithm for detecting the pupil and used knowledge about certain characteristics to discriminate the valid cornea SRs from other reflections from the surfaces glasses that could have been taken as cornea SRs [12]. In a previous study [13], they classified the reflection areas into four types and extracted the reflection parts. Ohtani *et al.*, proposed another method for solving the problems with reflections. Their method used two light sources and created a single image that incorporated differences between multiple images [14]. Ji *et al.*, proposed a system for monitoring driver vigilance that was based on eye gaze position and they tested users with or without glasses [15]. However, their method is limited because it does not consider cases where the illumination noises hide the pupil region completely, which is often the case in actual usage.

In order to overcome these problems, we propose a new gaze tracking method for use with users who are wearing glasses. In order to determine whether a user is wearing glasses or not, an image is captured in the initial stage with the camera set to the low exposure setting of the camera. The number of white pixels in the resulting image is counted. If the number of white pixels exceeds a certain threshold, it is determined that the user is wearing glasses because the SR noises usually occur on the surface of glasses. In this case, the four illuminators are controlled sequentially by turning them on and off and an image with minimal SR noises is obtained as a result. With this image, it is possible to locate the actual location of the pupil center and the positions of the four corneal SRs and eliminate the SR noises. Because one of the four illuminators is turned off, only three corneal SRs exist in the captured image. The invisible SR position is estimated based on the parallelogram shape that is defined by the other three SR positions. As a result, the gaze position can be calculated.

In a previous study [16], Böhme *et al.*, presented ideas for a gaze tracking system that is tolerant to eyeglasses. Their system is based on detecting whether the SRs from the eyeglass surfaces obscure the user's pupil and switching to a different set of illuminators at a different angle relative to the user and the camera. In this way, they proposed ideas for shifting the reflections away from the eyes or eliminating the reflections entirely. Although this method is similar to our method, it has not been implemented in an actual system. In our study, on the other hand, we construct an actual hardware system and the software algorithm for performing the performance measurements and analyses that are required in order to avoid the SRs from the eyeglasses. In addition, we propose a method for determining whether a user is wearing eyeglasses during the initial stage and use different algorithms based on whether the user is wearing or not wearing eyeglasses. We also propose a method for calculating the gaze position by estimating the invisible SR based on the parallelogram shape.

The remainder of this paper is organized as follows: Section 2 describes the details of the proposed method. The experimental results are presented in Section 3. The conclusions are discussed in the last section.

## 2. Proposed Method

### 2.1. Proposed Gaze Tracking System with the Device for Controlling Four Illuminators

**Figure 1.**
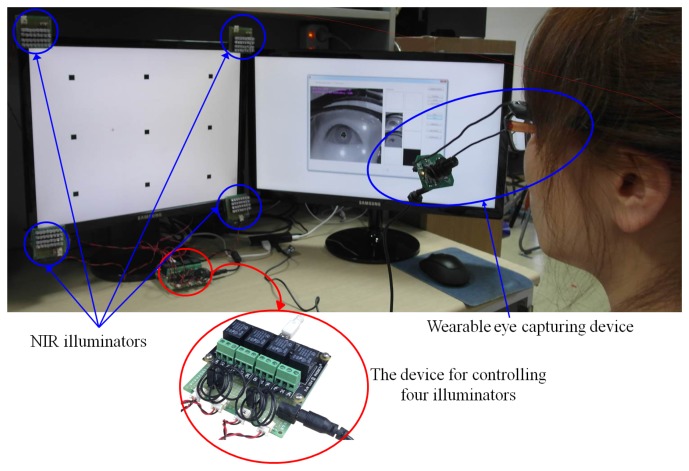
Proposed gaze tracking system.

In our study, we propose a new gaze tracking system with a device that controls four illuminators. Our gaze tracking system is based on a wearable device that includes a lightweight eye capturing camera [17] and is used in a desktop computer environment as shown in Figure 1. A conventional web-camera with a zoom lens of fixed focal length and a universal serial bus (USB) interface is used for the eye capturing camera. The field of view of the eye capturing camera (Figure 1) is −16.98°∼+16.98°.

Since the pupil area is usually distinctive in images that are captured by a near-infrared light (NIR) illuminator with a wavelength of 850 nm, the NIR cut filter (in the eye capturing camera) which passes the visible light is replaced with an NIR passing filter [17]. Four NIR illuminators are attached at the four corners of the monitor as shown in Figure 1 [17]. Each illuminator includes 32 NIR light emitting diodes (LEDs) with wavelengths of 850 nm. These four illuminators generate four corneal SRs and the quadrangle defined by these four SRs represents the monitor region [17].

We also constructed a device for controlling the four illuminators as shown in Figure 1. The device is constructed using a USB relay board [18] and it can be turned on and off selectively turn by controlling the power supply to the illuminator. That is, our gaze tracking program in a desktop computer determines whether the illuminator should be on or off and sends the command to the USB relay board via the USB interface.

### 2.2. Overview of the Proposed Method

**Figure 2.**
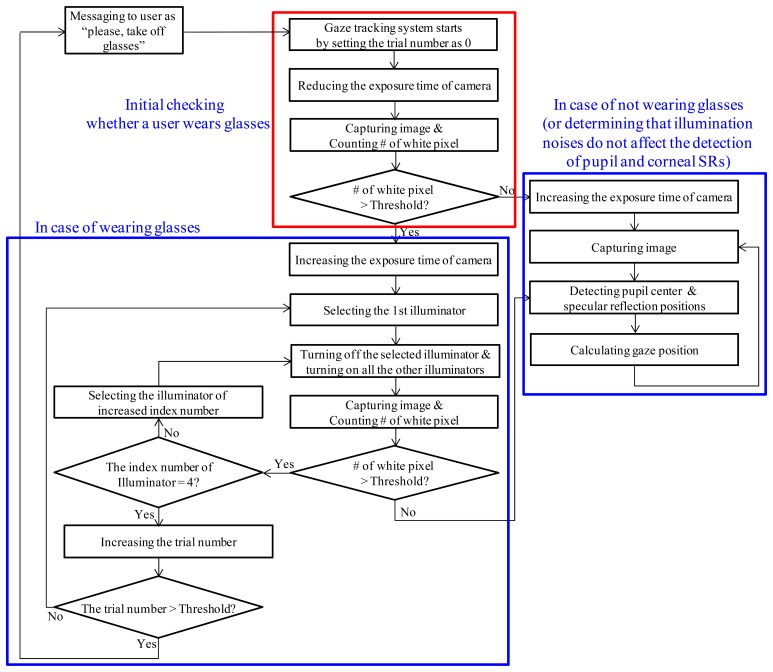
Overall procedure for the proposed gaze tracking method.

The overall procedure of the proposed method is shown in Figure 2. When the system starts, it performs the initial check in order to determine whether the user is wearing glasses or not.

**Figure 3.**
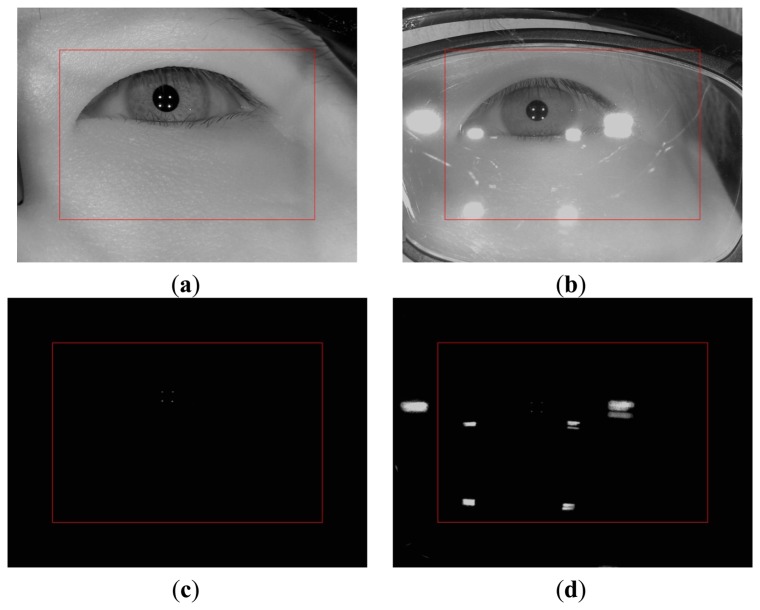
Eye images that were captured at various exposure times (**a**) Image of naked eye at the normal (unreduced) exposure time; (**b**) Image of eye with glasses at the normal (unreduced) exposure time; (**c**) Image of naked eye of (a) at the reduced exposure time; (**d**) Image of eye with glasses of (b) at the reduced exposure time.

In previous research, Wu *et al.*, proposed a method for detecting glasses using Haar and Gabor features based on boosting methods. However, they used the images where the entire face area and glasses were included for training and testing. In addition, both the Haar and Gabor features selected in the first boosting stage were detected in the area between the pair of eyes, which show that the nosepiece of the frame of the glasses was the important feature for detecting the glasses [19]. However, the nosepiece is not included in the image that is captured by our gaze tracking system, and the part of glasses frame may not be seen in the image, as shown in Figure 3b. Hence, the method in [19] cannot be used for our study.

Instead, the initial check that determines whether the user is wearing glasses or not is performed as follows. Firstly, the exposure time of camera is reduced and an image is acquired using the eye capturing camera in Figure 1. In general, if a user wears glasses, many reflections occur from the surfaces of the glasses as shown in Figure 3a,b. Since the shapes and sizes of the reflections vary, it is difficult to discriminate these reflections from reflections that are caused by the skin. In order to solve this problem, our system reduces the exposure time of the camera. Conventional cameras usually accumulate the light on the camera sensor during a 33.3 ms interval when the exposure time is set to 1/30 s. If the exposure time is reduced to 1/60 s, the time interval during which the camera accumulates the light is reduced to 16.7 ms (33.3/2). In general, the brightness of the reflections from the surfaces of the glasses is higher than the brightness of reflections from the skin because the reflection rate from the glasses is higher than that from the skin. As a result, the reflections from the skin cannot be seen when the exposure time of the camera is reduced as shown in Figure 3c,d. In this image, which was taken at a lower exposure time, the number of white pixels is counted within a predetermined area in the captured eye image (the red-colored box in Figure 3) because the eye is usually positioned in the restricted area by the device in Figure 1. If the number of white pixels exceeds a certain threshold (200), our system determines that the user is wearing glasses as shown in Figure 2.

**Figure 4.**
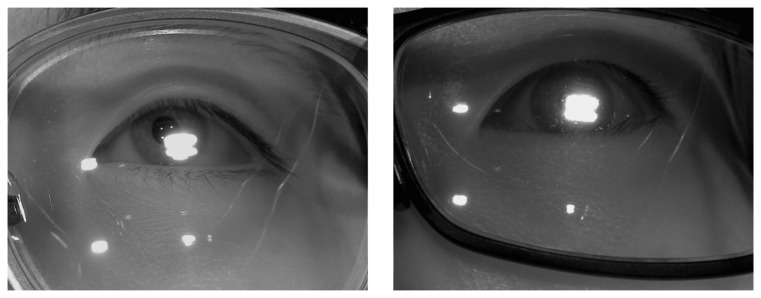
Examples of eye images where the reflections hide the pupil or the corneal SRs.

If it is determined that the user is wearing glasses, our system increases the exposure time (like Figure 3c,d) to the normal exposure time (like Figure 3a,b), and turns of the 1st illuminator. If all four of the NIR illuminators are on, they frequently cause reflections from the surface of the lens, as shown in Figure 4, and it is very difficult to detect the regions of the pupil and the four genuine corneal SRs.

As a result, our system turns the illuminators on and off sequentially. As shown in Figure 1, the four NIR illuminators are attached to the four corners of monitor and we designated the upper-left, upper-right, lower-left, and lower-right illuminators as the 1st, 2nd, 3rd, and 4th illuminators, respectively.

**Figure 5.**
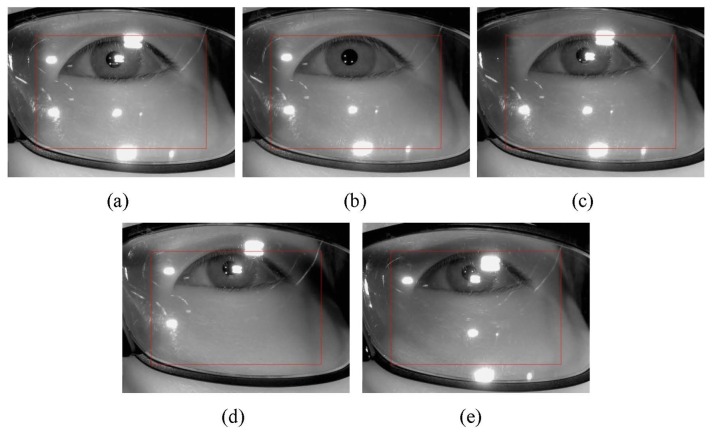
Examples of reflections as the illuminators are turned on and off in sequence (**a**) All four of the illuminators are on; (**b**) Only the upper-left illuminator (the 1st illuminator) is off; (**c**) Only the upper-right illuminator (the 2nd illuminator) is off; (**d**) Only the lower-left illuminator (the 3rd illuminator) is off; (**e**) Only the lower-right illuminator (the 4th illuminator) is off.

Our system turns off the 1st illuminator and captures an eye image. If the number of white pixel exceeds the threshold (15,000) within the pre-determined area of the eye image (the red-colored box of Figure 5), our system determines that many reflection noises still exist in the image with the 2nd, 3rd, and 4th illuminators. Thus, it selects a different illuminator to turn off. Accordingly, the 2nd illuminator is turned off and the other three illuminators (the 1st, 3rd, and 4th ones) are turned on. Another eye image is captured with these illuminators turned on and the number of white pixel is counted within the pre-determined area of the eye image (the red-colored box of Figure 5). If it exceeds the threshold (15,000), our system determines that many reflection noises still exist in the image and changes the illuminator that is turned off. The same procedures are repeated with the 3rd and 4th illuminators. If the number of white pixels is less than the threshold (15,000) in one of the resulting images, our system determines that the number of reflections is low enough because the corresponding illuminator has been turned off. At this point, the additional procedures for detecting the pupil and the corneal SR positions are performed and the final gaze position is calculated as shown in Figure 2. In order to cope with the worst case of an infinite loop (*i.e.*, the number of white pixels exceeds in threshold in all the cases), we include a stopping condition based on the number of trials as shown in Figure 2. If the trial number is greater than the threshold, our system displays a message to the user that says, “Please, take off your glasses”, and the gaze tracking system restarts. We set the threshold at 1.

**Figure 6.**
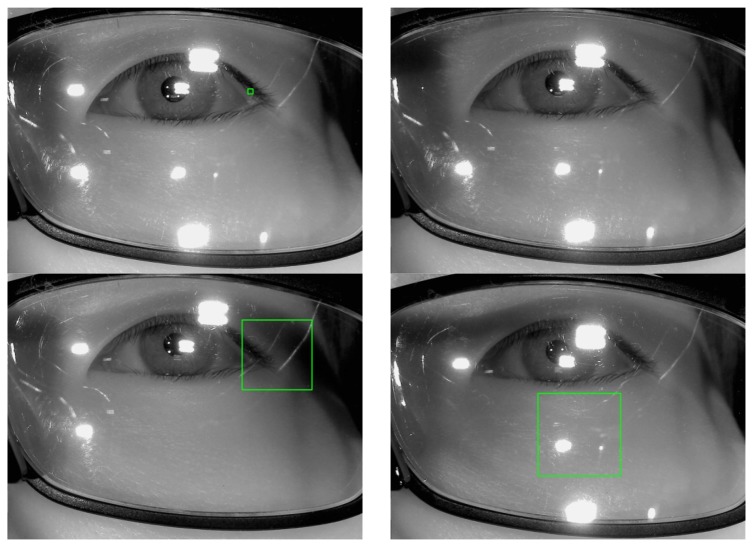
Examples of incorrect detections of the eye region while using the Adaboost eye detector for images that include reflections (We show the results for images in Figure 5a,c–e in the clockwise direction from the top-left image).

In order to accurately measure the effect of the reflections on the pupil region or the corneal SR, the number of white pixels should be counted in the detected eye region. However, a conventional eye detection algorithm based on the Adaboost method [20] does not give good performance for eye detection for images that include reflections as shown in Figure 6. The green box in Figure 6 represents the eye detection region and in the top-right image in Figure 6, there is no area that is detected by the Adaboost method. From these images, we can confirm that the Adaboost method cannot locate the eye region in images that include reflections inside the eye area. Thus, it is difficult to determine the actual eye region.

In our research, we used the Adaboost algorithm already trained, which are provided from OpenCV library (Version 2.4.2) [21], and we did not perform the additional procedure of training for the Adaboost algorithm. If we perform the training of the Adaboost with the sets including reflections like Figure 6, its performance of eye detection with the images including the reflections can be enhanced. However, the performance with the images of no reflection can be affected. In order to solve this problem, the training of the Adaboost should be performed with a lot of images with and without the reflections.

In our system, a user wears the gaze tracker device that is shown in Figure 1. Thus, the eye position in the captured eye image can be restricted within the predetermined area that is shown in Figures 3 and 5 (within the red-colored box). Based on this restriction, our system can determine whether the reflections have been removed by counting the number of white pixels in the pre-determined area of the image.

Figure 5 shows examples of reflections as the illuminators are turned on and off in sequence. Our system can determine that the image in Figure 5b is best for detecting the pupil and the corneal SRs and calculating the gaze position by comparing the number of white pixels in the pre-determined area (the red-colored box) of images in Figure 5a–e.

**Figure 7.**
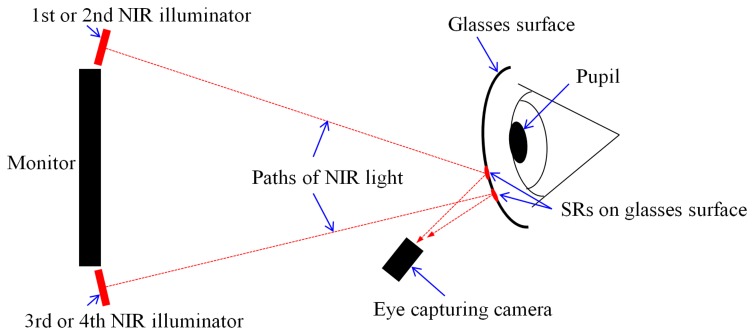
Conceptual diagram of the positions of the illuminators, the surface of the glasses, and the camera.

Because the camera in the wearable eye capturing device acquires the eye image below the eye, as shown in Figure 1, it is common for the eye region to be in the upper area of the glasses as shown in Figure 3b. In addition, based on the positions of the illuminators, the glasses, and the camera that are shown in the Figure 7, it is more likely for the SRs on the glass surfaces from the 1st (upper-left) and 2nd (upper-right) illuminators to be close to the eye region than it is for the SRs from the 3rd (lower-left) and 4th (lower-right) illuminators to be close as shown in Figures 3b and 13b. Thus, it is more likely to avoid the SRs by turning off the 1st or 2nd illuminators than it is to avoid the SRs by turning off the 3rd or 4th ones.

The procedure of turning off the 1st (upper-left) ∼4th (lower-right) illuminators with image capturing is sequentially performed as shown in Figure 2. For each image, if the number of white pixels is less than the threshold, the system determines that by turning off the corresponding illuminator, the number of reflections has been reduced. That is, if the image of Figure 5b satisfies the threshold for the number of white pixels, the systems stops the process of turning off illuminators and capturing images (thereby, not acquiring the images in Figure 5c–e). At this point, the procedures for detecting the pupil and corneal SR positions and calculating the gaze position are performed as shown in Figure 2. Consequently, based on these methods, the system determines that Figure 5b is the best image for the gaze detection process.

### 2.3. Detecting the Pupil Center and Corneal SR Positions

**Figure 8.**
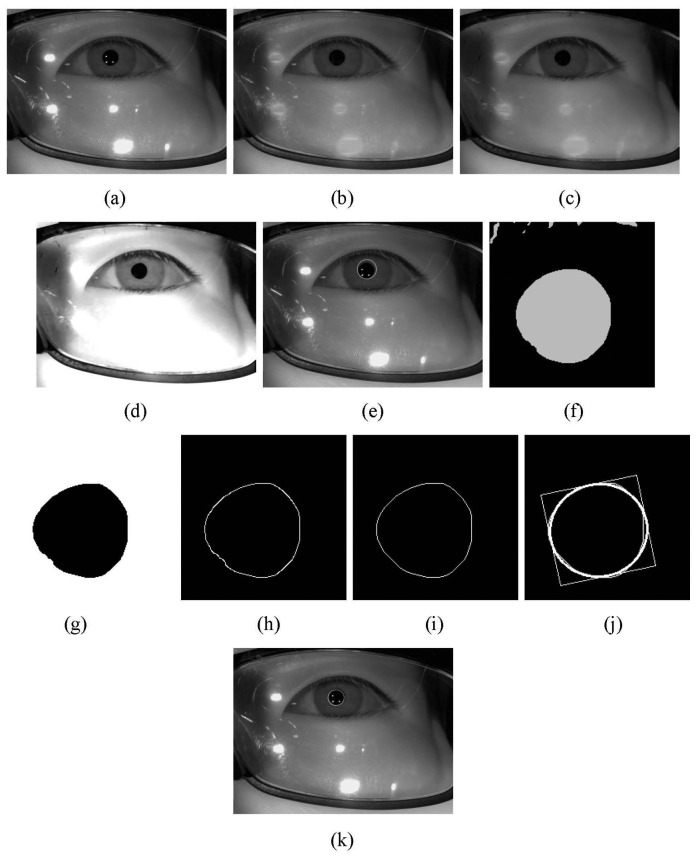
Examples from the pupil detection process. (**a**) Original image; (**b**) After erasure of the SR regions; (**c**) Image resulting from morphological operations; (**d**) Image resulting from histogram stretching; (**e**) Pupil area that is detected by the CED method; (**f**) Binarized image of the predetermined area (based on the detected pupil region) from (**d**); (**g**) Image resulting from morphological erosion and dilation of (**f**); (**h**) Result from component labeling and canny edge detection; (**i**) Result from the convex hull method; (**j**) Result from ellipse fitting; (**k**) Result of the pupil detection process.

If our system decides that a user is not wearing glasses or the reflections do not affect the detection of the pupil or the corneal SRs, the center of the pupil is located in the captured image as follows. Each part of the following pupil detection process (Figure 8) is novel, except for the circular edge detection (CED) (Figure 8e).

In general, SR areas have high pixel values and sharp changes in the gray values when compared to neighboring non-SR areas. This characteristic of sharp changes can cause errors in the pupil detection process. Thus, regions in the captured image that have bright pixels with gray levels that are higher than a threshold (200) are roughly estimated as SR regions. Then, these pixels are interpolated using their (left and right) neighboring pixels as shown in Figure 8b. As a result, the bright pixels have the characteristics of smooth changes in their gray values when compared to their neighboring ones. Then, the input image is processed using a morphological operation (the morphological opening is performed two times) in order to remove the reflections and group the regions with similar gray levels as shown in Figure 8c. In general, the pupil area is darker than other regions such as the iris, sclera, and skin. Thus, histogram stretching is performed as shown in Figure 8d in order to increase the differences in the pixel levels between the pupil and other regions. Then, the CED method is used to locate the approximate position of the pupil in the image as shown in Figure 8e [17,22,23]. However, the shape of the pupil is usually not perfectly circular. It is usually a more complicated shape. As a result, it is usually not possible to use the CED method to obtain an accurate detection of the pupil center. Thus, the restricted area of the image of Figure 8d based on the detected pupil center and radius by the CED is binarized as shown in Figure 8f.

Morphological erosion and dilation are performed on the binary image in order to remove the isolated reflections as shown in Figure 8g. Then, the image is processed using component labeling, canny edge detection, and the convex hull method [24] as shown in Figure 8h,i. Subsequently, the actual pupil area is detected using ellipse fitting (Figure 8j) [25] and the center of ellipse is designated as the center of the pupil as shown in Figure 8k.

The restricted region is binarized based on the detection of the pupil center. The regions whose sizes are smaller than the threshold (20) or bigger than the threshold (600) are removed by component labeling and size filtering processes. Then, the maximum four regions remained are selected, and the centers of the four regions are determined by calculating the geometric center of each region [17].

**Figure 9.**
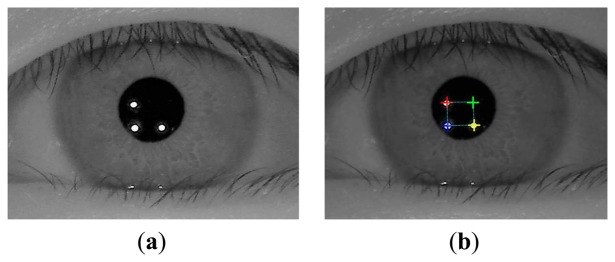
Example of estimating the unseen SR positions based on the parallelogram shape that is defined by the existing three SR positions (**a**) Original image (magnified eye region) of Figure 5b including three corneal SR regions; (**b**) Resulting image including four corneal SR regions (the upper-right corneal SR is estimated based on the parallelogram shape defined by the three existing SR positions).

In our system, one of the four NIR illuminators is turned off when the user is wearing glasses in order to avoid reflections as shown in Figure 2. Thus, only three corneal SRs exist in this case. Since the four NIR illuminators are attached at the four corners of monitor as shown in Figure 1, the quadrangle that is defined by the four corneal SRs represents the monitor region and the positions of these four SRs are required in order to calculate the gaze position. In order to solve this problem, the unseen SR position is estimated based on the parallelogram shape that is defined by the three existing SR positions that are shown in Figure 9, which is novel in our research.

**Figure 10.**
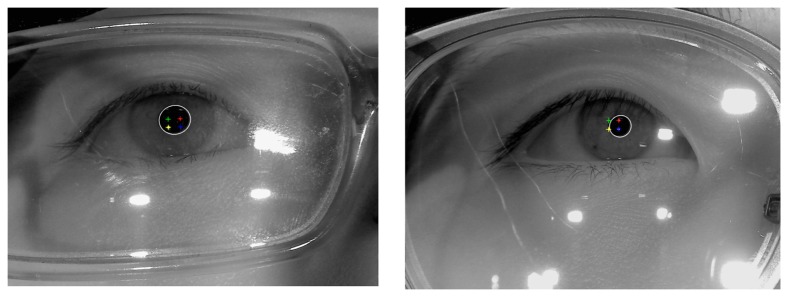
Examples of the results from the pupil detection and SR detection processes.

Figure 10 shows the results from the pupil detection and SR detection processes. The results in Figure 10 confirm that our method is able to locate the pupil and SR region correctly.

### 2.4. Calculating the Gaze Position

In order to calculate the gaze position in the monitor, we use a geometric transform method that is based on the locations of the center of the pupil and the centers of the four corneal SRs [17,26]. Then, the angle kappa is compensated for by the user-dependent calibration (each user gazes at the monitor center once during the initial stage) [17,27]. From that, the difference between the calculated gaze position and the monitor center is obtained, and it is compensated for calculating the final gaze position [17]. The resolution of the monitor that is used for the calibration is 1280 × 1024 pixels. Each user is instructed to gaze at the red (filled) circle. In order to induce the user's attention and increase the accuracy of the calibration accuracy, the diameter of the red circle is gradually reduced from 38 pixels to 30 pixels during the calibration process.

## 3. Experimental Results

The proposed method was tested on a desktop computer with an Intel^®^ Core™ i7 3.5GHz processor (Intel Corporation, Santa Clara, CA, USA) equipped with 8 GB RAM. Our algorithm was implemented using Microsoft Foundation Class (MFC) based C++ programming, the DirectX 9.0 software development kit (SDK), the library for controllable illumination devices, and the OpenCV library (Version 2.4.2) [21]. A 19-inch monitor with a resolution of 1280 × 1024 pixels was used.

**Figure 11.**
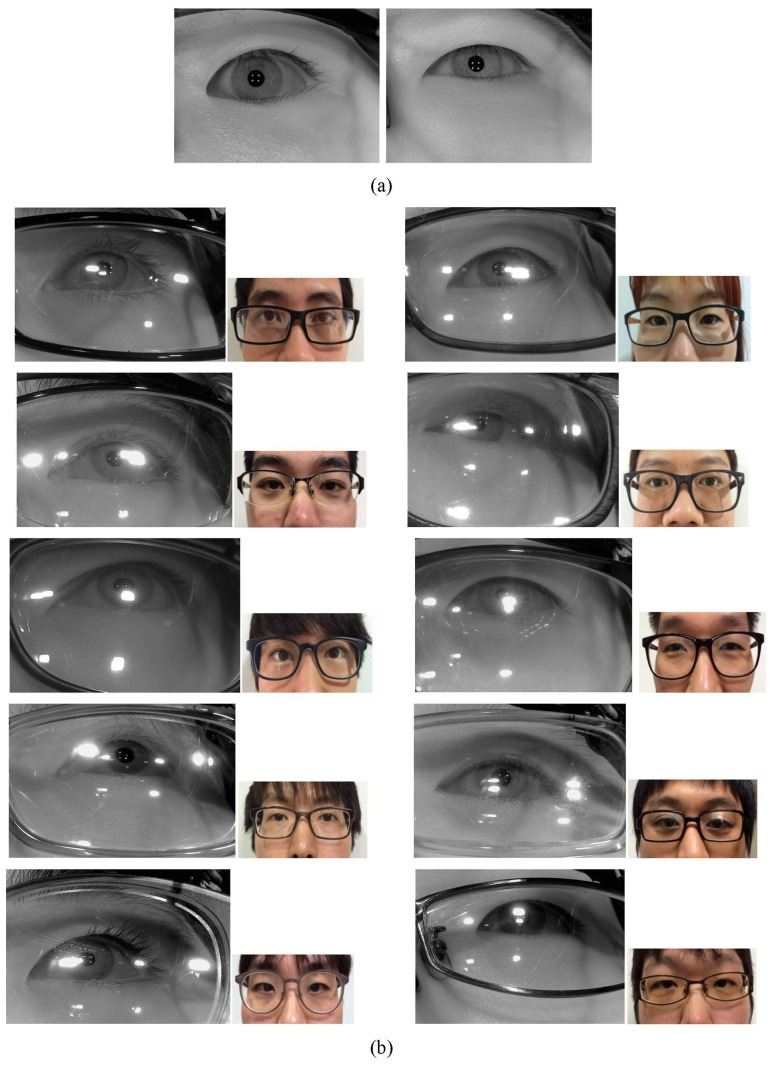
Examples of the images for experiments (**a**) Images of people not wearing glasses (**b**) Images of people wearing glasses (The top-left image is from user 1, the top-right image is from user 2, and the bottom-right image is from user 10 from Tables 1, 2, 4, 6, 8 and 10).

In the first test, we measured the accuracy of our system determining whether the users were wearing glasses or not (“initial checking whether a user wears glasses” of Figure 2). The experiments were performed with 400 images, which were captured from 20 persons. Each person tried 20 times. Our system captured an image using the low exposure setting of the camera during each trial for each of the test subjects. Out of the 20 participants, 10 wore glasses and the other 10 did not wear glasses. A total of 20 graduate students (whose ages were in the 20s to 30s range) volunteered to take part in the experiments without any payment. There were no restrictions during the selection of participants. Each the 10 persons brought their own glasses. Each pair of glasses that was worn by one of the 10 subjects was different from the others as shown in Figure 11b, and the number of glasses types is 10, consequently. Figure 11 shows the examples of captured images.

Detailed information about the glasses is shown in Tables 1 and 2. In Tables 1 and 2, users 1∼10 correspond to the users in Figure 11b and Tables 1, 2, 4, 6, 8 and 10. In Tables 1 and 2, the spherical strength of the glasses is shown as “S-XXX” where the number “XXX” represents the diopter of the lens. Information about astigmatism of the lenses is also shown in Tables 1 and 2. The larger number in “C-XXX” represents the highest degree of astigmatism of the lenses. And we show the kind of lens and glasses frame. We also subjectively evaluated the quality of the glasses surfaces as high, medium, or low as shown in Tables 1 and 2.

We define Type 1 and Type 2 errors for measuring the accuracy of the proposed method. A Type 1 error means that the test subject was wearing glasses, but it was incorrectly determined that they were not wearing glasses. A Type 2 error signifies that the test subject was not wearing glasses, but it was incorrectly determined that they were wearing glasses.

**Figure 12.**
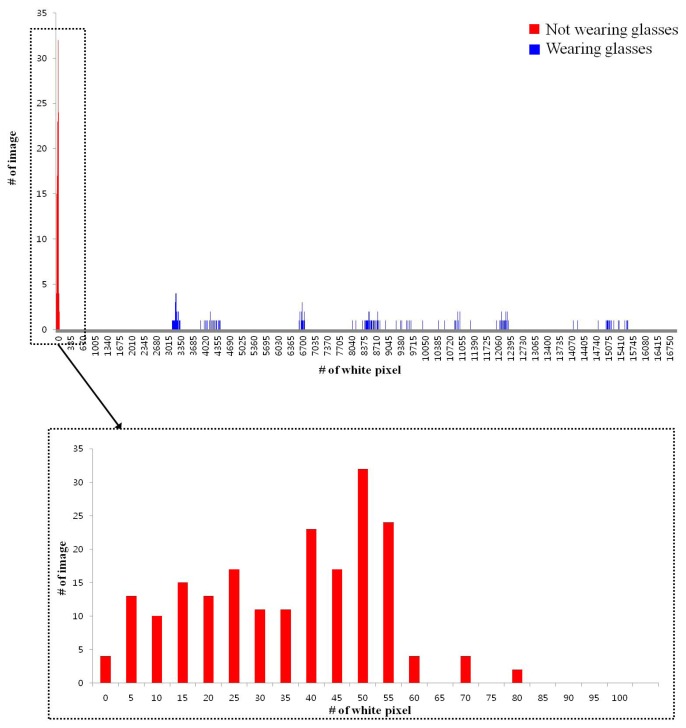
Two histogram distributions for not wearing and wearing glasses in terms of the numbers of white pixels.

The experimental results showed that the rate of Type 1 and Type 2 errors was 0%. That is because the two distributions of wearing glasses and not wearing glasses do not have any overlapped area as shown in Figure 12.

**Figure 13.**
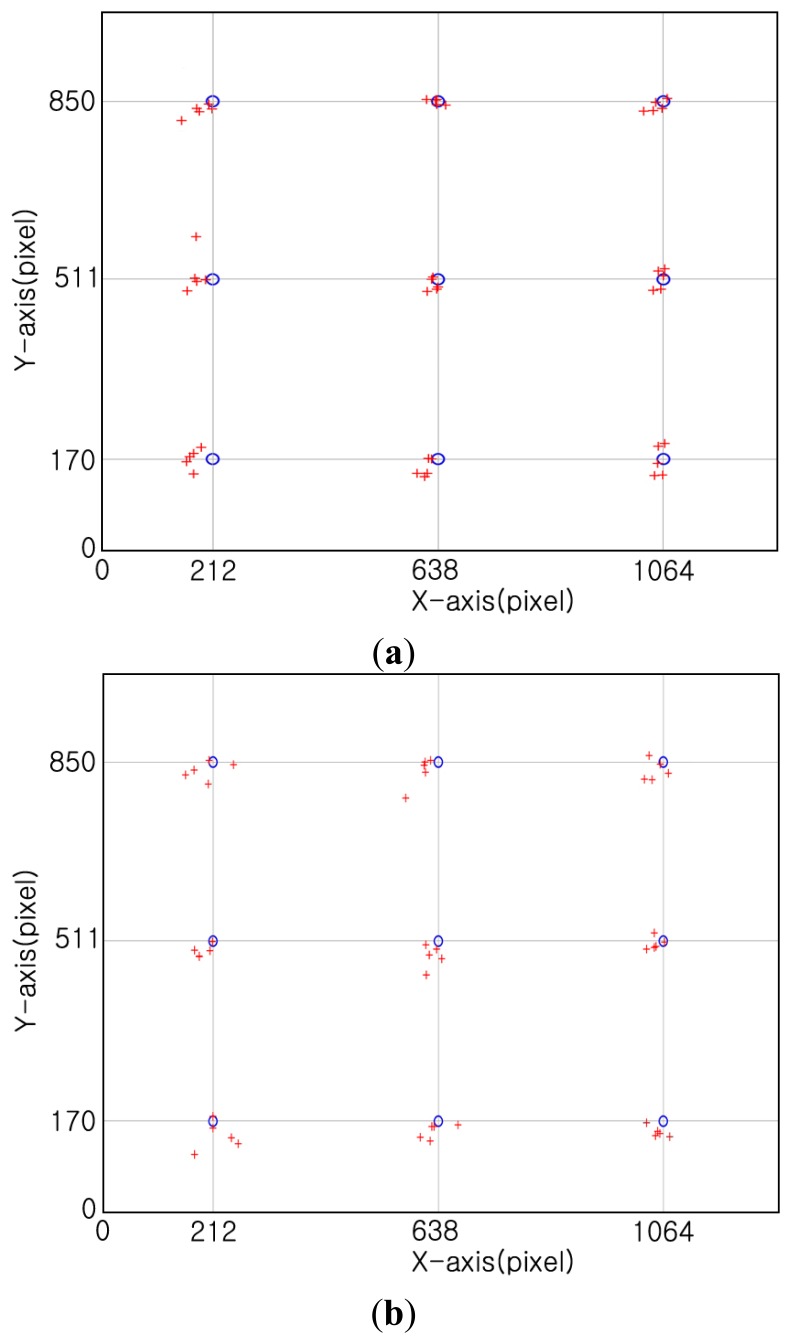
Example of the calculated gaze positions based on the nine reference points (five trials of one person) (the “°” symbols signify the reference points and the “+” signs are the detected gaze points) (**a**) User is not wearing glasses (**b**) User is wearing glasses.

In the second test, we measure the error of our gaze tracking system. The distance between the monitor and the eyes of participants is about 85 cm. We tested with 20 users. Each person is requested to gaze at 9 reference points on the monitor as shown in Figure 13. Among 20 persons, 10 people wore glasses, and the other 10 people did not wear glasses. These experiments were repeated five times per each person. Thus, each person gazes at the 45 gaze positions (9 reference points × 5 times). Each person is instructed to look at the monitor center for the initial user calibration (see Section 2.4), and see the nine reference points (of Figure 13) five times. Except for these, no instruction was given. All the participants were allowed to move their head freely.

The error of gaze detection is measured as the unit of ° based on the difference between the reference and the calculated gaze points. The difference means the angular disparity of two vectors (one is from the pupil center to the reference point, and the other is from the pupil center to the calculated point).

Tables 3 and 4 display the gaze detection errors that occurred for users who were not wearing glasses and for user who were wearing glasses, respectively. As shown in Tables 3 and 4, the error for the former group (0.70°) is almost same to that of the latter group (0.70°). As a result, we conclude that our gaze tracking system works irrespective of whether the users are wearing glasses or not. The reason why the errors were greater for users 5 and 8 than for other users in Table 3 was that these users failed to gaze at the exact center point of the monitor during the initial calibration of the angle kappa that is described in Section 2.4.

As shown in Table 4, the lowest gaze errors were obtained with users 2–4 and the highest gaze errors were for users 5 and 10. Based on these results and the analyses of the characteristics of the glasses of Tables 1 and 2, we found that there is no relationship between the properties of the glasses and the accuracy of the gaze detection process.

When the images in Figure 11a with b are compared, it is apparent that the images of people wearing the glasses include much larger SRs from the glass surfaces. Some of the SRs hide the pupils or the corneal SRs. Nevertheless, as shown in Tables 3 and 4, the average gaze detection errors for users who are not wearing the glasses is about 0.70° and it is the same for users who are wearing glasses.

For the stochastic analysis of the experimental results, we compared the errors in Table 3 to the errors in Table 4 and used the *t*-test [28,29] to establish the confidence levels. For the two tailed *t*-test with the null-hypothesis (the total average error of Table 3 is same to that of Table 4), we obtained a *p*-value as 0.9862. Because the p-values are greater than 0.01 (*i.e.*, confidence level of 99%), the null-hypothesis fails to be rejected [28] and we can conclude that the total average error for the former case (users not wearing the glasses in Table 3) is almost identical to the total average error for the latter case (users wearing glasses in Table 4) with a confidence level of 99%. Thus, we can conclude that the proposed method solves the problem with the SRs from the surfaces of glasses hiding the pupil or the corneal SRs and that the proposed method obtains accurate gaze positions irrespective of whether the user is wearing glasses or not.

Tables 5 and 6 show the gaze detection errors for each of the nine reference points in Figure 13. The upper-left, upper-center, upper-right, middle-left, middle-center, middle-right, lower-left, lower-center, and lower-right reference points in Figure 13 are the gaze positions for 1–9 in Tables 5 and 6, respectively. As shown in Tables 5 and 6, the gaze detection errors at the four positions (close to the four monitor corners) of 1, 3, 7 and 9 seem to be larger than others. The reason for this result is that each user gazes at the center of the monitor during the user-dependent calibration for the angle kappa that is described in Section 2.4. This calibration process does not provide sufficient information about the angle kappa when the user gazes at positions that are close to the corners of the monitor.

In addition, we include the differences between the gaze detection errors along the X and Y coordinates in Tables 7 and 8. The results in the tables show that the gaze detection errors along the X coordinate were similar to those along the Y coordinate.

Figure 13 shows examples of the calculated gaze positions based on the 9 reference points. The resolution of the monitor is 1,280 × 1,024 pixels and the point that each user was supposed to gaze at is shown as a black (filled) circle with a diameter of 30 pixels.

In Figure 13, the nine reference points are displayed as blue (blank) circles in order to enhance the distinctions between the calculated gaze points (red cross marks) and the reference points. During the experiment, black (filled) circles were actually used as the reference points.

It is difficult to compare our method to previous methods because different hardware systems and different methods were used for detecting the pupil and the SRs. As a result, we have opted to compare the accuracy of proposed method with the accuracy of [30]. In [30], the cross-ratio-based method was used in conjunction with vanishing points in order to calculate the gaze position. In order to construct a fair comparison, the same method was used for the initial calibration, the process for controlling illuminators, the process for detecting the pupil and the SRs. As shown in Tables 3, 4, 9 and 10, the experiment confirms that the accuracy levels of the proposed method are higher than those from the previous method [30].

**Figure 14.**
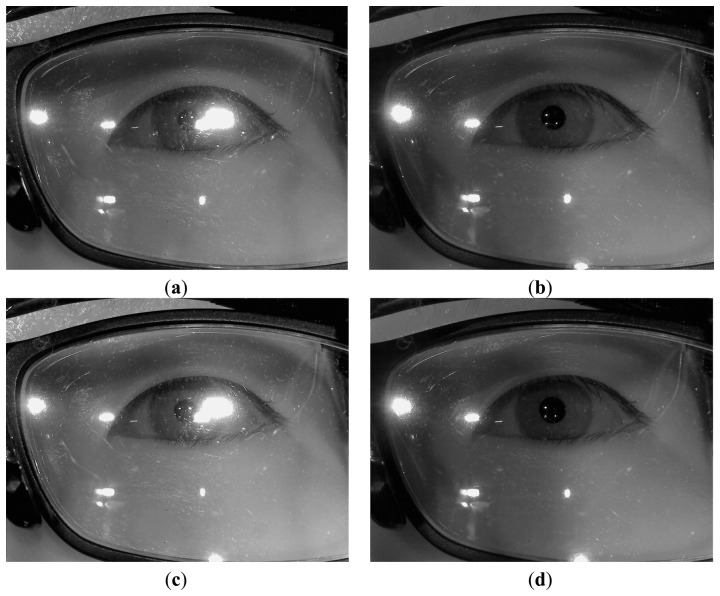
Images for cases where the outer fluorescent light was on or off (**a**) All of the NIR illuminators are on and the outer fluorescent light is on; (**b**) Only the upper-left illuminator (the 1st illuminator) off and the outer fluorescent light is on; (**c**) All of the NIR illuminators are on and the outer fluorescent light is off; (**d**) Only the upper-left illuminator (the 1st illuminator) is off and the outer fluorescent light is off.

The captured eye images were not affected by outer lighting conditions because the NIR cut filter, which passed visible light into the eye capturing camera, was replaced with an NIR passing filter [17] and NIR illuminators were used. As shown in Figure 14, the image brightness and the status of the pupil, corneal SRs, and SR noises in Figure 14a,b (outer light on) are almost similar to those in Figure 14c,d (outer light off).

Table 11 shows the processing times from our gaze tracking system for each sub-module. We do not include the average processing time (about 84.1 ms) in Table 11 for the initial check for determining whether the user is wearing glasses because it is performed once only during the initial stage, and it is not performed again after that. The average processing time (about 0.83 s) for turning off the illuminator and checking for reflections (the procedures from the bottom-left (blue) box in Figure 2) is also not included in Table 11 because it is only performed once.

As shown in Table 11, the processing time for the case where the user is wearing glasses is similar to that for the case where the user is not wearing glasses. When the user is wearing glasses, the step of turning off the illuminator and checking for reflections (the procedures of the bottom-left (blue) box of Figure 2) is also performed and as a result, the processing time is increased by as much as 0.83 s. However, because this step is only performed once, the processing time for wearing glasses is nearly identical to the processing time for the case where the user is not wearing glasses after this process is completed as shown in Table 11. Based on the average total processing time of 63.72 ms, we conclude that our system can be operated at the speed of about 15.7 frames/s (1000/63.72).

**Figure 15.**
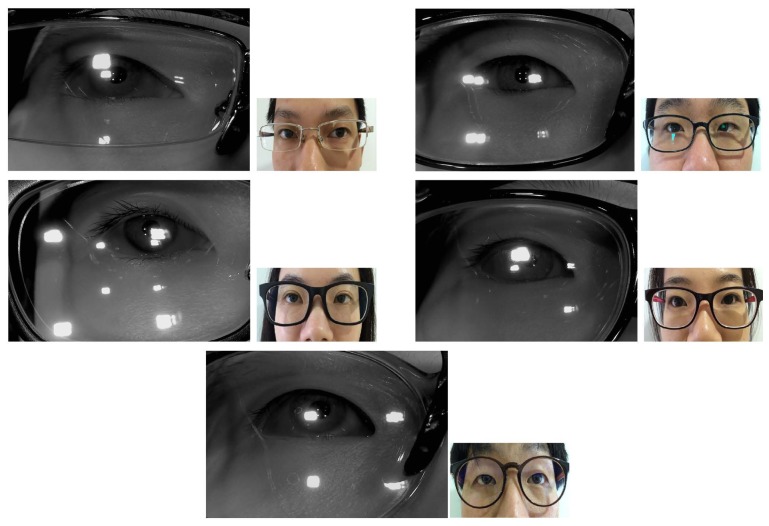
Images of people wearing glasses (The top-left image is from user 11, the top-right image is from user 12, and the bottom image is from user 15 from Tables 12 and 13).

In order to analyze the influences of the properties of the glasses on the results in a more systematic fashion, we included five additional participants (whose ages are in the 20s) with glasses that were different from those of users 1–10 in Figure 11b and Tables 1, 2, 4, 6, 8 and 10. The images of people wearing glasses are shown in Figure 15. The characteristics of the glasses of the additional users (users 11–15) are shown in Table 12 and the gaze detection accuracy levels for these users are shown in Table 13. Since the glasses of user 12 do not include the functionality of correcting nearsightedness (myopia), there is no information on the spherical strength of the glasses.

As shown in Table 4, the lowest gaze errors were obtained with users 2–4, and the highest gaze errors were for users 5 and 10. When comparing users 2–4 with users 5 and 10 in Tables 1 and 2, the (spherical) strength of the glasses of user 2 is similar to that of user 10. Astigmatism correction is not included in the glasses of user 3, while that is included in users 2 and 4. The lens type of user 5 is a convex lens while that of user 10 is a concave one. The types of glasses frames of users 2–4 are different (plastic, aluminum, and wood ones, respectively). As shown in Table 13, the lowest gaze error was obtained with user 15, and the highest gaze error was for user 14. When comparing user 14 with user 15 in Table 12, the lens types of users 14 and 15 are similar concave ones. The types of glasses frames of users 14 and 15 are also similar plastic ones. From this, we found that there is no relationship between the properties of the glasses and the level of the accuracy of the gaze detection process.

We can think that the glasses surface of lower quality can usually produce more reflections and the consequent error of gaze detection increases. However, the qualities of glasses surface of users 2∼4 are low while those of users 5 and 10 are high, as shown in Tables 1 and 2. From that, we found that there is also no relationship between the quality of the glasses' surface and the level of the accuracy of our gaze detection method.

The accuracy levels and frame rates of commercial systems are typically very high. The frame rates of these systems are usually very high due to the use of expensive, high speed cameras. As a result, the overall costs of these systems are very high. They also tend to be very bulky. For example, the size of Tobii TX300 Eye Tracker is 55 × 24 × 6 cm^3^ [31]. However, the cost and size of the proposed system are very low because the system is based on a low-cost web-camera. Although the accuracy of the commercial system was reported as 0.5°, the accuracy level for users with glasses was not reported [31].

The average processing time for the proposed system is 63.72 ms as shown in Table 11, but most of the processing time is concerned with detecting the pupil (Figure 8) in the 1600 × 1200 pixel image. In order to reduce the processing time, we sub-sampled the original image, obtained an image with 800 × 600 pixels, and performed the pupil detection (Figure 8) using the sub-sampled image. Experimental results with the data from users 11–15 in Tables 12 and 13 showed that the average processing time was reduced by about 23.47 ms (42.6 Hz). The level of accuracy for gaze tracking with the revised method was almost 0.64° as shown in Table 13, which was similar to the level of accuracy in Tables 3 and 4.

## 4. Conclusions

In this paper, we have proposed a new method for tracking the gaze of a user who is wearing glasses. This method is based on a scheme for controlling the illuminator and estimating the unseen SR position based on the parallelogram shape. Through experiments with the data from 20 test subjects, we were able confirm that our system was effective regardless of whether the test subject was wearing glasses or not.

In order to reach a higher level of accuracy during the gaze tracking process, a high resolution image of the eye should be acquired as shown in Figure 3a. A high resolution camera with a zoom lens is required in order to accomplish this. The viewing angle of the camera in a gaze detection system with a zoom lens will be very small. As a result, a non-wearable (non-head-mounted) gaze tracking system should include functionality for panning and tilting in order to track the eye region based on the natural movements of the user's head. However, this kind of functionality will cause the size and cost of the system to increase. Therefore, we use a head-mounted (wearable) gaze tracking system that is lightweight and inexpensive. Our system allows the user to move naturally because the camera in our system is attached to the user's head and moves with the user.

The image of the eye that is captured by the camera in the head-mounted system is not distorted when the user moves because the camera moves with the user. The image of the eye from the non-wearable gaze tracking system, on the other hand, can be distorted by head movements, which can reduce the accuracy of the gaze detection process. Non-wearable gaze tracking systems are usually more convenient for the user than head-mounted systems, but the inconvenience of our system is reduced through the use of a lightweight frame and a lightweight web-camera. As a result, our system can be used in various applications that require a compact and inexpensive, yet accurate gaze tracking system. It can be used in desktop computer environments for monitoring the web-surfing patterns of users, measuring the effects of advertisement during web-browsing, and also during driver training or pilot training. We plan to test our system in various environments, including outdoors, in a future study. We also plan to research methods for increasing the processing speed of our system.

## Figures and Tables

**Table 1.** The characteristics of the glasses for users 1–5 from Tables 1, 2, 4, 6, 8 and 10.

**The characteristics of** **the glasses**	**User 1**	**User 2**	**User 3**	**User 4**	**User 5**
**(Spherical) strength of** **glasses (Right eye/Left eye)**	(S-275/S-275)	(S-350/S-325)	(S-250/S-250)	(S-475/S-350)	(S-0.0/S-0.25)
**Astigmatism (Right eye/Left** **eye) (Astigmatism angle)**	No	Yes (C-50/ C-50) (180)	No	Yes (C-0.75/ C-0.75) (180)	Yes (C-70/ C-45)(180)
**Concave or convex lens**	Concave lens	Concave lens	Concave lens	Concave lens	Convex lens
**Type of glasses frame**	Plastic	Plastic	Aluminum	Wood	Plastic
**Quality of glasses surface**	Medium	Low	Low	Low	High

**Table 2.** The characteristics of the glasses for users 6–10 from Tables 1, 2, 4, 6, 8 and 10.

**The characteristics of** **the glasses**	**User 6**	**User 7**	**User 8**	**User 9**	**User 10**
**(Spherical) strength of** **glasses (Right eye/Left eye)**	(S-275/S-225)	(S-550/S-400)	(S-250/S-250)	(S-450/S-400)	(S-350/ S-350)
**Astigmatism (Right eye/Left** **eye) (Astigmatism angle)**	No	Yes (C-150/ C-200) (180)	No	No	Yes (C-50/ C-50) (180)
**Concave or convex lens**	Concave lens	Concave lens	Concave lens	Concave lens	Concave lens
**Type of glasses frame**	Plastic	Plastic	Plastic	Plastic	Aluminum
**Quality of glasses surface**	Medium	Low	Low	Medium	High

**Table 3.** The errors in gaze detection for cases where the users were not wearing glasses (units: °).

**Trial number and** **average error**	**User 1**	**User 2**	**User 3**	**User 4**	**User 5**	**User 6**	**User 7**	**User 8**	**User 9**	**User 10**
**1st trial**	0.62	0.90	0.65	0.62	0.88	0.30	0.67	1.0	1.0	0.44
**2nd trial**	0.39	0.65	0.63	0.69	0.71	0.35	0.56	0.83	0.85	0.81
**3rd trial**	0.66	0.63	0.87	0.35	0.98	0.43	0.79	1.04	0.73	0.66
**4th trial**	0.5	0.87	0.77	0.56	0.98	0.46	0.57	1.01	0.98	0.64
**5th trial**	0.37	0.77	0.58	0.81	1.06	0.4	0.50	1.00	0.61	0.97
**Average**	0.51	0.76	0.70	0.61	0.92	0.39	0.62	0.98	0.83	0.7
**Total average error of 10 users**	0.70									

**Table 4.** The errors in gaze detection for cases where the users were wearing glasses (units: °).

**Trial number and** **average error**	**User 1**	**User 2**	**User 3**	**User 4**	**User 5**	**User 6**	**User 7**	**User 8**	**User 9**	**User 10**
**1st trial**	0.59	0.46	0.76	0.51	0.82	0.78	0.56	0.84	0.93	0.72
**2nd trial**	0.66	0.65	0.47	0.60	0.79	0.77	0.68	0.85	0.90	0.90
**3rd trial**	0.84	0.55	0.53	0.74	0.58	0.66	0.74	0.32	0.67	0.84
**4th trial**	0.64	0.87	0.69	0.57	0.82	0.75	0.74	0.87	0.76	1.00
**5th trial**	0.66	0.53	0.61	0.59	0.92	0.52	0.98	0.86	0.43	0.51
**Average**	0.68	0.61	0.61	0.60	0.79	0.70	0.74	0.75	0.74	0.79
**Total average error of 10 users**	0.70									

**Table 5.** The gaze detection errors for each of the nine reference points when the users were not wearing glasses (units: °).

**Gaze position**	**1**	**2**	**3**	**4**	**5**	**6**	**7**	**8**	**9**

**User**									
**User 1**	0.78	0.58	0.51	0.81	0.32	0.34	0.61	0.19	0.4
**User 2**	0.53	0.78	0.81	0.47	0.52	0.73	0.86	1.11	1.08
**User 3**	0.4	0.59	0.72	0.46	0.45	0.64	0.91	1.09	1.05
**User 4**	0.49	0.27	0.56	0.54	0.28	0.78	0.48	0.66	1.4
**User 5**	0.27	0.54	0.74	0.98	0.54	1.46	1.14	0.99	1.62
**User 6**	0.45	0.44	0.36	0.37	0.27	0.45	0.4	0.31	0.44
**User 7**	0.91	0.44	0.61	0.93	0.31	0.48	0.8	0.46	0.55
**User 8**	0.96	0.76	0.9	0.95	0.45	0.95	1.21	1.14	1.48
**User 9**	0.86	0.61	1.33	0.81	0.8	0.62	0.96	0.4	1.11
**User 10**	0.39	0.73	0.64	0.34	0.77	0.75	0.4	0.78	1.53
**Average**	0.6	0.57	0.72	0.67	0.47	0.72	0.78	0.71	1.07

**Table 6.** The gaze detection errors for each of the nine reference points when the users were wearing glasses (units: °).

**Gaze position**	**1**	**2**	**3**	**4**	**5**	**6**	**7**	**8**	**9**

**User**									
**User 1**	1.28	0.78	0.73	1.18	0.18	0.43	0.75	0.43	0.36
**User 2**	0.79	0.59	0.55	0.53	0.67	0.38	0.71	0.72	0.57
**User 3**	0.7	0.64	0.7	0.55	0.44	0.71	0.65	0.43	0.68
**User 4**	0.96	0.4	0.75	0.75	0.49	0.72	0.11	0.17	1.07
**User 5**	1.23	0.84	0.77	0.95	0.3	0.85	0.75	0.61	0.77
**User 6**	0.89	0.63	0.94	0.66	0.43	0.87	0.62	0.41	0.82
**User 7**	0.72	0.5	0.95	0.84	0.44	0.59	1.09	0.61	0.91
**User 8**	0.78	0.28	1.13	0.95	0.3	0.8	1.02	0.56	0.93
**User 9**	0.99	0.5	0.55	0.7	0.77	0.75	0.94	0.57	0.88
**User 10**	0.82	0.84	1.03	0.87	0.67	0.6	0.48	0.83	0.97
**Average**	0.92	0.6	0.81	0.8	0.47	0.67	0.71	0.53	0.8

**Table 7.** The errors of gaze detection along X and Y-coordinates in cases where the users were not wearing glasses (units: °).

**X or Y coordinate**	**X**	**Y**

**User**		
**User 1**	0.33	0.31
**User 2**	0.49	0.51
**User 3**	0.39	0.5
**User 4**	0.3	0.46
**User 5**	0.57	0.64
**User 6**	0.23	0.27
**User 7**	0.25	0.62
**User 8**	0.52	0.7
**User 9**	0.59	0.46
**User 10**	0.41	0.49
**Average**	0.408	0.496

**Table 8.** The gaze detection errors along the X and Y-coordinate in case where the users were wearing glasses (units: °).

**X or Y coordinate**	**X**	**Y**

**UserX or Y coordinate**		
**User 1**	0.52	0.34
**User 2**	0.4	0.41
**User 3**	0.35	0.43
**User 4**	0.34	0.46
**User 5**	0.5	0.51
**User 6**	0.46	0.44
**User 7**	0.55	0.4
**User 8**	0.75	0.53
**User 9**	0.5	0.44
**User 10**	0.57	0.42
**Average**	0.494	0.438

**Table 9.** The gaze detection errors from the previous method [30] for users who were not wearing glasses (units: °).

**Trial number and** **average error**	**User 1**	**User 2**	**User 3**	**User 4**	**User 5**	**User 6**	**User 7**	**User 8**	**User 9**	**User 10**
**1st trial**	0.82	1.71	1.71	1.49	2.42	1.32	1.34	1.9	1.6	1.77
**2nd trial**	0.56	1.78	1.7	1.8	2.21	1.34	1	1.66	1.56	1.54
**3rd trial**	0.99	1.84	1.47	1.42	2.31	1.38	1.17	1.63	1.83	1.8
**4th trial**	0.62	1.53	1.34	1.52	2.43	1.41	0.96	1.5	1.98	1.56
**5th trial**	0.58	1.44	2.02	1.54	2.47	1.04	0.96	1.49	1.57	1.7
**Average**	0.71	1.66	1.65	1.55	2.37	1.3	1.09	1.64	1.71	1.67
**Total average error of 10 users**	1.54									

**Table 10.** The gaze detection errors from the previous method [30] for users who were wearing glasses (units: °).

**Trial number and** **average error**	**User 1**	**User 2**	**User 3**	**User 4**	**User 5**	**User 6**	**User 7**	**User 8**	**User 9**	**User 10**
**1st trial**	1.29	1.82	1.25	1.54	2.12	2.29	1.52	1.61	1.1	1.81
**2nd trial**	1.38	1.16	1.62	1.99	1.89	1.89	1.42	1.74	0.94	2.44
**3rd trial**	1.47	1.25	1.78	2.05	2	2.07	1.57	2.49	1.27	1.88
**4th trial**	1.33	1.86	1.72	1.64	1.91	2.06	1.46	1.78	0.96	2.07
**5th trial**	1.53	1.24	1.69	2.18	2.04	1.71	1.65	1.63	2.24	1.31
**Average**	1.4	1.47	1.61	1.88	1.99	2	1.52	1.85	1.3	1.9
**Total average error of 10 users**	1.69									

**Table 11.** The processing times for sub-modules in the gaze tracking system (units: ms).

**Cases of not wearing** **or wearing glasses**	**Pupil** **detection**	**Corneal SR** **detection**	**Calculating gaze** **position**	**Total** **processing time**	**Average total** **processing time**
**Not wearing glasses**	59.06	3.83	0	62.89	63.72
**Wearing glasses**	60.14	4.40	0	64.54	

**Table 12.** The characteristics of the glasses of users 11–15.

**The characteristics of** **the glasses**	**User 11**	**User 12**	**User 13**	**User 14**	**User 15**
**(Spherical) strength of glasses** **(Right eye/Left eye)**	(S-200/S-300)	N/A	(S-125/S-350)	(S-625/S-425)	(S-25/S-25)
**Astigmatism (Right eye/Left eye)** **(Astigmatism angle)**	No	Yes (C-50/ C-50) (90)	No	Yes (C-50/C-125) (180: Left/160: Right)	No
**Concave or convex lens**	Concave Lens	Concave lens	Concave lens	Concave lens	Concave lens
**Type of glasses frame**	Stainless	Plastic	Plastic	Plastic	Plastic
**Quality of glasses surface**	High	Medium	High	Medium	High

**Table 13.** The gaze detection errors for users who were wearing glasses (units: °).

**Trial number and** **average error**	**User 11**	**User 12**	**User 13**	**User 14**	**User 15**
**1st trial**	0.67	0.63	0.61	0.66	0.55
**2nd trial**	0.64	0.64	0.62	0.67	0.59
**3rd trial**	0.64	0.51	0.65	0.67	0.52
**4th trial**	0.7	0.58	0.68	0.79	0.62
**5th trial**	0.68	0.8	0.64	0.65	0.5
**Average**	0.67	0.63	0.64	0.69	0.56
**Total average error of 5 users**	0.64				
